# Supplementary material for: Are carbon emissions trading and green financial instruments synergistic? -Comprehensive quantitative research based on content analysis
Source: PLoS One. 2024 Mar 7;19(3):e0298601. doi: 10.1371/journal.pone.0298601 (PMC10919601; doi:10.1371/journal.pone.0298601)
Supplement: S1 Data — (ZIP) [file pone.0298601.s001.zip › Data/地级市Z_F工作报告.zip!/┴╔─■/╜⌡╓▌╩╨/└┤╫╘╣1⁄2╓┌║┼í╛┬φ┐╦╩2╛▌═°í┐_2006.docx]

各位代表：

　　现在，我代表市政府向大会报告工作，请予审议，并请政协委员提出意见。

　　一、“十五”主要成绩和“十一五”发展目标

　　进入新世纪以来的五年是不平凡的五年，全市人民在省委、省政府和市委的正确领导下，坚持以邓小平理论和“三个代表”重要思想为指导，全面落实科学发展观，紧紧抓住老工业基地振兴和沿海地区开放的双重机遇，深入实施工业立市、以港兴市、开放牵动三大战略，聚精会神搞建设，一心一意谋发展，全市经济社会保持了快速健康发展的良好势头，较好地完成了“十五”计划确定的各项目标和任务。

　　“十五”期间，全市地区生产总值年均增长１３．８％，超过预期４．８个百分点。预计２００５年地区生产总值比２０００年增长近一倍，人均生产总值达到１５００美元。全社会固定资产投资累计完成３５２．７亿元，比“九五”时期增长一倍，年均增长１６．２％。地方财政一般预算收入年均增长１２．３％。社会消费品零售总额年均增长１２．３％。实际利用外资累计完成４．９亿美元。出口创汇累计完成１２．６亿美元，年均增长４１．２％。城市居民人均可支配收入和农民人均纯收入分别比２０００年增加３１５４元和１４３５元，年均增长９．２％和１０．２％。

　　各位代表，过去的五年，是我市改革开放不断取得突破，综合实力显著增强，人民群众得到实惠最多的五年；是城区面貌发生明显改观，基础设施建设取得重大进展，城市功能进一步完善的五年；是社会各项事业和谐发展，精神文明和民主法制建设取得可喜进步的五年。在这里，我代表市政府，向在各个领域和岗位上辛勤劳动、做出贡献的全市人民，向给予我们支持与监督的人大代表、政协委员、各民主党派、工商联和各人民团体、社会各界人士，向大力支持我们工作的驻锦人民解放军和武警部队表示崇高的敬意和衷心的感谢！

　　过去五年的实践，进一步深化了我们对锦州经济社会发展规律的认识，积累了许多宝贵经验。

　　第一，必须坚持科学的发展战略。思路决定出路。市委提出的工业立市、以港兴市和开放牵动三大战略切合实际，“三年大变样、十年大振兴”目标初见成效，我们必须一以贯之地贯彻执行，坚持不懈地为之努力。

　　第二，必须坚持又快又好地发展。高效益是检验工作质量的最终标准。只有以高效益为前提的快发展，才能扭转落后局面，提高竞争能力，加快推进经济社会的和谐发展。

　　第三，必须坚持深化改革扩大开放。改革解决矛盾，开放增添活力。只有依靠不断解放思想，深化改革，扩大开放，调动各方面的积极性、创造性，才能形成加快发展的强大动力。

　　第四，必须坚持把人民利益放在第一位。不断改善人民生活是执政为民的根本体现。只有真正解决好事关百姓利益的就业再就业、社会保障、棚户区改造等现实问题，才能赢得人民群众的支持和拥护。

　　第五，必须坚持奋力拼搏的工作作风。良好工作作风是事业成功的关键。当前锦州经济社会的发展，更是时刻离不开奋力拼搏精神的弘扬与光大。

　　这些经验对于我们实现“十一五”期间全市经济社会发展目标必不可少，至关重要。

　　“十一五”期间是我市改革开放和现代化建设的关键时期，也是实现“三年大变样、十年大振兴”目标的重要阶段。根据市政府提请本次会议审议的《锦州市国民经济和社会发展第十一个五年规划纲要草案》，“十一五”期间我市经济和社会发展的主要标志是：确保经济增长速度持续超过全省平均水平，确保城市人均可支配收入和农民人均纯收入增长幅度超过全省平均水平。具体目标是：到２００７年，基本构筑起区域性中心城市总体框架，实现“三年大变样”；到２０１０年，基本实现老工业基地振兴，地区生产总值实现８００亿元、年均增长１５％以上，人均生产总值超过３０００美元，单位生产总值能源消耗比“十五”末期降低２０％左右，全社会固定资产投资年均增长２３％，地方财政一般预算收入与生产总值协调增长，城市居民人均可支配收入和农民人均纯收入分别达到１４２００元、６０００元，城镇登记失业率控制在５％以内，为建设辽西沿海经济区中心城市奠定坚实基础。

　　实现“十一五”目标，是党中央、国务院和省委、省政府的重托，也是全市人民的殷切希望。我们将以高度的使命感和责任感，全力推进辽西沿海经济区中心城市建设，不断开创各项工作的新局面，努力把“十一五”的宏伟蓝图变成美好的现实。

　　二、２００５年工作回顾

　　２００５年是我市经济社会发展取得重大进展的一年。在市委的正确领导下，全市上下坚持以邓小平理论和“三个代表”重要思想为指导，全面贯彻落实科学发展观，按照市委九届八次、九次全会精神，围绕“百姓腰包鼓起来、保障体系建起来、城乡环境美起来、社会治安好起来、文化氛围浓起来”的具体工作目标，以昂扬向上的精神风貌和奋力拼搏的工作斗志，进一步深化改革扩大开放，大力实施工业倍增、农业升级、城市南扩、建设商贸中心、打造文化名城、构筑和谐锦州六大举措，保持了经济社会的快速健康协调发展，较好地完成了市十三届人大三次会议确定的各项任务。

　　－－国民经济快速增长。初步预计，全市生产总值实现３８４亿元，按可比价格计算，增长１６．５％，其中一二三产业增加值分别完成８８亿元、１４５亿元和１５１亿元，分别增长６％、２０％和１６％。地方财政一般预算收入实现１７．３亿元，增长１６．７％。

　　－－发展动力不断增强。社会消费品零售总额实现１３７亿元，增长１３％。全社会固定资产投资实现９２亿元，增长２５．２％，其中工业固定资产投资完成３４亿元。实际利用外资新口径完成６６４７万美元（老口径为７６９３万美元），增长６９．６％；实际利用域外资金３２亿元，增长３８．３％。预计出口创汇５．２亿美元，增长４３．９％。

　　－－临港经济势头良好。港口吞吐量预计达到３０００万吨，增长２２．２％；集装箱吞吐量达到２０．１万标箱，增长９５．１％。开发区实际利用外资２６３３万美元，增长４１．６％；规模以上工业总产值实现２５．２亿元，增长４３．９　％。

　　－－人民生活继续改善。城镇劳动力就业实名制安置９．３万人，城镇登记失业率控制在５．７％以内。城市居民人均可支配收入８８６２元，增长１１％；农民人均纯收入３７３０元，增长９．７％。

　　这些成绩的取得，标志着＂三年大变样、十年大振兴＂开局良好，也标志着我市经济社会进入了新的快速发展时期。

　　一年来，我们主要抓了以下八方面工作：

　　（一）坚持改革开放，经济发展活力进一步显现

　　坚持把扩大开放作为增强发展后劲、增添发展活力的重要途径，大力实施开放牵动战略，努力在全市营造重视开放、支持开放、参与开放的浓厚氛围。特别是国办３６号文件下发后，深入学习贯彻文件精神，先后召开锦州港、开发区、凌海市、国有企业以及多领域等专题对外开放工作会议，全市上下掀起了第三轮扩大开放的新高潮。坚持以日韩、港台、珠三角、长三角等国家和地区为主，有针对性、有重点地走出去招商，香港投资说明会、广州投资说明会、深圳家乡人恳谈会暨投资说明会等招商引资活动取得圆满成功。日电铁合金、中海集装箱、进永精密锻造、美联桥汽车零部件等一批项目落户锦州，真空开关管、大唐锦州热电厂、阿迪达斯运动鞋、路易达孚大豆深加工、沃尔玛等一批在谈项目进展顺利。锦州港对外开放龙头牵动作用凸显，港口基础设施建设进一步完善，３０万吨级油品泊位、两个５万吨级集装箱专用泊位、进港主航道拓宽等工程竣工投入使用。开发区开放先导示范作用进一步增强，锦州湾核心区域开发全面启动，投资４．３亿元的西海国际工业园开工建设。凌海对外开放步伐进一步加快，娘娘宫申报省级开发区工作正在进行，临海工业带规划初步形成。

　　全方位深化改革，努力消除制约发展的体制性、机制性障碍。农村税费改革、粮食流通体制改革、土地延包等政策得到落实，粮食直补和良种补贴资金全部发放到户，９９％的村完成了二轮土地延包工作。制定出台了深化国有企业改革、加强国有资产管理等政策意见，１５０个国企辅助单位和企业办社会职能机构完成了主辅分离，华光、焊条、叉车等８５户国有企业实现改制。市直３９４家事业单位机构分类定位和落实编制＂实名制＂工作初步完成。第三次行政审批制度改革圆满结束，行政审批事项削减到２６７项，窗口办结率提高到５０％。文化体制改革有序推进。农村信用社改革取得阶段性成果。

　　（二）推进工业倍增，工业经济实力进一步提升

　　科学分析工业经济现状和未来三年发展机遇，制定并全力实施了工业经济三年倍增计划，工业经济保持了较快发展速度。规模以上工业总产值完成４７９．２亿元，增长３６％；增加值完成９９．３亿元，增长２８．９％。全市产值５亿元以上企业达到１２户，其中元成、沈宏、华宇、华龙４户企业步入１０亿元以上企业行列。新开工建设１０００万元以上项目１１７个，其中５０００万元以上２４个，亿元以上１３个。中石油锦州石化公司７５０万吨炼量扩能改造、锦恒公司年产２０万套安全气囊、华昌硅公司８英寸硅单晶棒、华光集团８０ＫＶ陶瓷真空开关管、锦开电器等３６个项目相继竣工投产。１４个工业国债项目顺利推进，攀钢钛业３万吨钛白粉等１０个项目开工建设，其中小牛血去蛋白注射液、苯磺酸等４个项目实现部分投产。新争取省技改贴息项目２６个，财政贴息资金达到４５８２万元。三个优势产业、两个新兴产业发展迅速，四个传统产业结构进一步调整优化，＂三二四＂产业格局更加清晰。加大政策扶持、金融服务、依法保护和环境治理力度，努力为民营企业创造宽松的发展环境。民营工业增加值实现１４７．８亿元，税金１１．５亿元。地方工业发展势头迅猛，规模以上县（市）区属工业企业总产值实现１９７．５亿元，增长５９．４％。

　　（三）实施农业升级，农村发展环境进一步优化

　　结合我市实际，全面启动实施了农业结构、经营模式和增长方式升级计划。农林牧渔业总产值预计实现１６５亿元，增长６％。粮食总产量达到２０１万吨，增长７万吨，再创历史新高。新增有机、绿色食品认证４０个，无公害农产品认证４个。新建现代农业科技园区５０个，畜牧小区５５个，肉羊改良站１００个。禽产品、林产品、水产品等十大农业产业化经营体系不断完善壮大，规模以上农业产业化龙头企业达到１１７户。农业生产条件进一步改善，完成水土流失治理１８．６万亩，增加有效灌溉面积１１．３　万亩，农业机械化水平达到５７％。人工植树造林４８．２万亩，森林覆盖率达到２２．３％。落实治理海水倒灌方案，大小凌河扇地防海水入侵一期工程已经完成，二期工程正在抓紧报批，整个工程有望在今年１０月完工。中心渔港建设竣工在即，南凌一级渔港即将开工建设。积极抢修应急度汛工程，顺利实现安全度汛。农村剩余劳动力实现转移就业５．２万人。

　　面对突如其来的禽流感疫情，在党中央、国务院的亲切关怀和省委、省政府的正确领导下，以及人民解放军和武警官兵的大力支持下，全市上下众志成城，按照＂加强领导、密切配合，依靠科学、依法防治，群防群控、果断处置＂的２４字方针，快速发动、科学应对，　７个市级防控工作组和２０００多名专业技术人员、公安干警、医务人员、机关干部深入防控一线，与黑山、北宁、南站新区等县（市）区的广大干部群众一道合力攻坚，取得了防控禽流感工作的决定性胜利。

　　（四）加速城市南扩，基础设施建设进一步完善

　　老城区改造和管理力度进一步加大。新建大修道路１１５条，其中改造小街小巷１０１条。投入１５１３．６万元，分两批对２５０栋楼、１０３万平方米非物业住宅区环境进行了彻底整治。１００条＂摸黑路＂改造工程全部完工。新增供暖面积８０万平方米、燃气用户１万户、公交线路２条。站前广场等重点地段实施交通整治，交通秩序明显好转。制定了城市管理三年规划，通过实行门前三包、网格化管理、聘请城市管理监督员等办法，城市管理水平明显提高。全面开展了争创国家卫生城、国家环保模范城活动。城市南扩＂十大工程＂全面启动。渤海大道、市府广场、女儿河带状公园、凌南道路网、滨海公路工程开工建设，凌南市场建设和第三医院组建工作进展顺利，南山核心区绿化任务接近尾声，实验学校竣工并按期开学，凌南棚户区拆迁工作稳步推进，拆迁面积达到４０多万平方米。重点基础设施建设项目进展顺利。完成了士英街排水改造工程和女儿河４公里截流干管工程。污水处理厂、垃圾处理场、粪便无害化处理厂建成，图书馆工程即将竣工投入使用。完成村村通油路工程３２７．５公里，６４个村新通油路。１０２国道等公路大中修工程全部完工。高天地方铁路增能改扩建工程完成２２００延长米，铁路年综合运输能力达到１５００万吨。全市建筑工程质量总体水平稳居全省前列，渤海大学图书馆工程获国家优质工程大奖，率先实现辽西地区＂鲁班奖＂工程零的突破。

　　（五）建设商贸中心，商贸财税实力进一步增强

　　稳步提升传统商贸业，加快发展现代服务业，全力打造服务辽西的区域性商务中心、购物中心、集散中心、物流中心。消费市场进一步繁荣活跃，元都大酒店等投入运营，新华购物广场等即将竣工开业，天宇商贸中心等先后开工建设。物流业发展步伐加快，恒大物流保税仓库通过验收开始运营；华联物流配送网络发展到赤峰、本溪、阜新等８个城市；渤海物流一期工程完工，中海集团北方物流、满洲里神怡鲜储公司等陆续入驻园区。建立健全市县两级农产品质量安全监测体系，实现了城区所有封闭市场速测机构的全覆盖；建立公开透明的农产品质量安全信息发布制度，通过媒体向全社会发布检测结果；创建无公害农产品销售示范市场，食品市场准入制度进一步落实，有力保证了市民食品安全。会展业有新的发展，成功举办了锦州市首届房展会、第八届中国古玩艺术品博览会和第九届中国（锦州）北方农业新品种、新技术展销会。

　　加强税费征管，出台了《关于进一步完善市与县（市）区财政体制的决定》，禁止滥拉税源，严厉打击偷逃骗税行为，税收征管秩序进一步规范。通过压缩一般性支出，削减专项支出和公用经费，以及多方筹集资金，重点保证了工资发放、社会保障和救灾救济等支出。特别是市本级在财力十分紧张的情况下，通过调整支出结构，保证了国家公务人员地方性补贴政策的落实。积极争取到省开发银行５亿元贷款额度，缓解了失业保险、城市基础设施建设资金短缺问题。金融业运行平稳，资产进一步优化，改革取得新的进展。全市银行业金融机构本外币各项存款余额达到５１１．９亿元，增长１１．６％。保险业快速发展，保险金额达到１０亿元，增长３５．６％。

　　（六）打造文化名城，社会各项事业进一步繁荣

　　积极推进科技自主创新，３９项科技成果通过省、市鉴定，其中８项达到国际先进水平，１５项达到国内领先水平。争取国家和省级科技计划项目７３项，科技三项经费实际到位１６５６万元，支持科技项目９１个。全市高新技术产业产值预计完成９６亿元，增长２７．８％；增加值完成２４．３亿元，增长５５．４％。科技进步对全市经济增长贡献率达到５１％。顺利组织了＂辽西五市首届毕业生择业洽谈大会＂等各类人才交流会。成功举办了古塔春节庙会、笔架山海会、国际民间文化节和周末文化广场等大型文化活动，组织了《凤鸣辽西》电视春节文艺晚会及几百场文艺演出。广播电视覆盖率达到９９．８％，开通数字电视４００余户。文化市场体系进一步健全，文艺演出、音像电子、古玩艺术品等九大行业发展快速，年销售收入达到３亿元。进一步整合旅游资源，打造精品线路，辽沈战役纪念馆＂红色旅游＂品牌在省内外叫响。旅游总收入实现３０．４亿元，增长４０．１％。撤并中小学７９所，建设九年一贯制（寄宿制）学校８所，农村中小学布局更趋科学合理；普通高中教育实现跨越式发展，中等职业教育规模不断扩大；完成＂宏志班＂招生４５０人，解决外来打工人员子女入学５１４人。省十运会会徽、会歌、吉祥物、主题语和开幕日期均已确定，场馆改造、竞赛安排、宣传、筹资等项工作顺利推进。积极开展义县农村新型合作医疗试点工作，加大流行性出血热等传染病的防治力度，公共卫生体系进一步完善。人口出生率和自然增长率分别控制在７．１７‰和２．２６‰以内。圆满完成了第一次全国经济普查各项任务。档案信息、人防地震等工作取得新的进展。

　　（七）构筑和谐锦州，安定祥和氛围进一步浓厚

　　大力开展就业再就业工作，全年实名制培训再就业人员２．４万人，为下岗失业人员发放小额贷款２８９２万元、减免税费２３３７万元，１７３２９户＂零就业家庭＂全部实现了至少有１人就业。全力实施社会保险扩面工作，＂五险合一＂的社会保障体系全面建立。养老、失业、医疗、工伤、生育保险参保人数分别达到３１．６万人、３８．７万人、４０．７万人、２０万人和８．１万人。养老保险金按时足额发放，保障水平有所提高。归集住房公积金２．７亿元，年归集总量创历史新高。进一步完善社会救助体系，城区低保对象大病救助保险制度运行良好，全年救助５５０人次，赔付３３０万元，被省民政厅誉为＂锦州模式＂，得到国家民政部肯定。全市城市低保对象达到７万余人，基本实现了应保尽保；建立了农村低保制度，保障对象达到４．６万人。社区＂一站四室＂建设全面启动，１３６个社区＂一站四室＂面积平均达到２６７平方米，给广大社区居民带来极大方便。清理网上拖欠工程款６．４亿元、农民工工资３６５万元。深入开展了军民携手绿化南山、民营企业拥军等军民共建和拥军优属活动，双拥工作成果显著。民族宗教工作取得新的进展。坚持扶贫工作经常化，安排救助走访金６４０万元；深入实施整村推进扶贫开发工程，２８个村、近３万人得到扶助。为支持疫区人民抗击禽流感，全市掀起了捐款热潮，慈善总会共收到捐款１０００多万元，充分体现了锦州人民团结互助的崇高精神。

　　贯彻落实新《信访条例》，继续实施信访公开接待日和主要领导包案制度。安排信访领导接待日４４次，处理信访案件３８起；各级领导共包案６１５件，已办结５２６件，办结率８６％、息访率７０％。认真开展了公安＂大接访＂活动，社会治安形势明显好转。１１０、１１９、１２２＂三台合一＂建设取得重大进展，平安县（市）区创建工作全面展开，新型治安防控体系初步建立，全市刑事案件和治安案件大幅下降，打击刑事犯罪绩效考核全省名列第一。建立了突发公共事件应急指挥保障体系，编制了总体预案、基本案、专项预案和保障计划。人民调解和法律援助工作取得新的成效，＂四五＂普法工作顺利完成。加强生产、交通和消防安全管理，安全生产形势总体平稳。

　　（八）加强政府建设，依法行政能力进一步增强

　　认真开展保持共产党员先进性教育活动，实现了＂两促进、两提高＂。自觉接受人大法律监督、工作监督和政协民主监督，坚持政府与人大代表、政协委员联系制度。全年办理市人大议案２件，人大代表建议和政协提案４８６件，办复率、满意率分别达到１００％和９６％。积极落实行政执法责任制，认真做好行政复议和应诉工作，依法行政得到加强。全面推进政务公开工作，建立了新闻发布会制度，及时向社会发布各类信息，提高了政府工作的透明度。深入开展＂机关作风建设年＂活动，制定了公务员行政效能投诉处理办法、行政效能监察规定等制度，对不作为、慢作为、乱作为等问题进行了公开有效的监督和处理。出台了《关于严肃机关工作纪律的四项规定》，坚持对餐饮娱乐场所明察暗访，发现、处理违纪问题８起，给予７人公开曝光和纪律处分。＂求真务实、雷厉风行、令行禁止、严细成风＂的十六字要求深入落实，公务员的行政行为进一步规范，快速反应、务实高效的行政指挥系统逐步建立，以＂锦州港精神＂、＂实验学校速度＂、＂南山绿化干劲＂等为代表的＂想干事、真干事、干成事＂的工作作风和行政氛围日益浓厚。

　　回顾一年的工作，我们必须清醒地看到，全市经济社会发展中还面临着一些困难和问题。一是农业生产方式落后。无论是种植业、养殖业，还是农产品加工业，都不同程度地存在着生产方式落后问题。特别是去年发生的禽流感疫情，将我市家禽养殖业饲养分散、密度过大、畜禽混养等传统落后的生产方式问题暴露无遗，农业升级迫在眉睫。二是个别工业企业亏损严重。工业生产虽然呈现出强劲的增长态势，但由于原油、煤、电等重要基础原材料和能源价格上涨，导致石化公司、东港电力、热电公司等大户企业出现了不同程度的亏损。三是国有企业改革进展缓慢。国有企业改革工作受政策、改革成本等多方面因素影响，进展速度缓慢，改革任务十分艰巨。四是财政收入水平有待进一步提高。由于产业结构不合理、工业总量偏小、县域经济发展缓慢等因素影响，全市税收收入增长还不够快，财政状况还不宽裕。上述问题都需要我们在今后的工作中采取有力措施加以解决。

　　三、２００６年工作安排

　　２００６年是实施＂十一五＂规划的开局之年，也是实现＂三年大变样＂目标的关键一年。做好今年全市工作，对实现＂三年大变样＂目标，为＂十一五＂期间经济社会发展奠定坚实基础，具有十分重要的意义。

　　２００６年经济社会发展的总体要求是：以邓小平理论和＂三个代表＂重要思想为指导，认真贯彻省委九届十一次全会暨全省经济工作会议和市委九届十次全会精神，坚持以科学发展观统领经济社会发展全局，围绕建设辽西沿海经济区中心城市的目标，以锦州湾整体开发为突破口，加快实施工业倍增、农业升级、城市南扩、建设商贸中心、打造文化名城、构筑和谐锦州六大举措，着力提高经济增长质量和效益，努力推动全市经济社会又快又好地发展。

　　２００６年经济社会发展的主要目标：地区生产总值增长１５％，其中一二三产业增加值分别增长８％、１８％和１６％；地方财政一般预算收入增长１２％；全社会固定资产投资增长２５％；实际利用外资增长２０％，域外招商引资增长２３％，实际出口创汇增长１０％；社会消费品零售总额增长１２％；物价总水平涨幅控制在３％以内；城市居民人均可支配收入和农民人均纯收入分别增长１０％和８％；城镇登记失业率控制在５．７％以内。

　　为实现上述目标，今年要着重抓好以下十项工作：

　　（一）突出锦州湾整体开发，加速沿海经济带建设

　　深入贯彻省委、省政府沿海城市经济带＂三点一线＂　开发战略，按照我市＂一港、一区、一带、一面＂的具体构想，加大锦州湾整体开发力度，加速打造锦州沿海经济带。

　　做强做大锦州港。加大投入力度，积极推进２０５、２０６、５０２泊位、粮食现代物流和进港主航道浚深等工程的施工进度。大力开拓腹地货源市场，确保年内吞吐量进一步大幅度增长。调整完善第三、四港池的总体规划和特大型散货泊位、煤炭专业化泊位、油品专用泊位建设方案。加快西部港区一期开发建设筹备工作进程，开工建设西部港区。

　　加速开发区提档升级进程。积极做好国家级开发区申报工作，保税物流中心争取年内申报成功。调整完善开发区总体规划，将开发区规划面积扩大至２８平方公里。高标准做好西海国际工业园、白马综合工业园和白沙湾海滨新区建设规划，全力推进园区基础设施建设和项目摆放步伐。

　　加快凌海工业带建设。以娘娘宫申报省级开发区为契机，抓紧启动＂三区二园一基地＂（物流仓储区、滨海别墅区、特色海珍品养殖区，临港工业园、水产品加工工业园，水产品批发、加工、运销集散基地）和凌南、双羊、娘娘宫、建业、大有、阎家、大凌河口等７个工业园区建设，大力发展农产品深加工、金属冶炼、油田配套、精品海盐等项目，加速形成临海工业带。

　　加大沿海开发对各县（市）区全面发展的带动作用。进一步整合资源，积极促进锦州港、开发区、凌海市所属沿海工业园区和工业项目的延伸衔接，加大沿海经济对其它地区发展的牵动辐射作用，努力实现黑山、北宁、义县等县（市）区和沿海经济带的统筹联动发展。

　　（二）强力推进项目建设，加快工业倍增进程

　　发展重大项目。深入实施＂工业倍增＂计划，以重大项目牵动工业经济总量、规模的扩张。加大７５个投资３０００万元以上续建新建项目的建设力度，重点推进中石油锦州石化公司１５万吨异丙醇、８万吨苯乙烯、中海集装箱、华光金属化陶瓷壳、新日硅硅单晶片、元成１０万吨高果糖、西洋特肥５０万吨复合肥等项目。建立工业发展振兴基金，努力争取国债和省财政贴息资金，切实加大对重大工业项目的支持力度。规模以上工业增加值增长２５％，工业固定资产投资增长２０％以上。

　　优化结构布局。继续按照＂三二四＂格局调整工业结构，积极吸引民间资本发展石油化工、汽车零部件、农产品深加工、新材料、电子信息、机械装备制造等行业，形成产业集群，拉长产业链条。整合资源，激活要素，盘活存量，加快汤河子工业区振兴步伐。实施品牌战略，引导和扶持企业创建发展自有知名品牌。加强对工业经济运行中重大问题的研究和调控，从整体上提高工业企业产品竞争能力，切实提高工业企业效益。

　　加快国企改革。筹集国企改革专项基金，建立完善的国有资产管理体制，规范国企改制和产权交易，确保国有资产保值增值。取消省属以下国有企业改制的股权限制，拓宽改革成本的筹措渠道，妥善处置改制企业的债权债务，做好职工安置工作。重点抓好金城、铁合金、燃气等３８户企业改制工作，确保年内国有企业改制任务全面完成。

　　壮大民营工业。重点支持元成、沈宏等企业做强做大，鼓励有实力的企业跨地区、跨行业发展，努力培育具有国际竞争力的企业集团。支持民营企业加大投入，帮助中小企业与工业主导产业对接。积极扶持民营科技企业，鼓励民营企业参与国有企业改革。加大培训工作力度，着力打造高素质民营企业家队伍。民营经济增加值占地区生产总值比重达到６０％以上。

　　（三）全面实施农业升级，建设社会主义新农村

　　认真贯彻党中央、国务院＂建设社会主义新农村＂的各项要求，全面落实农业升级１０大指标，加快农业结构、经营模式和增长方式的升级。

　　调整优化农业结构。提高高效农作物比例，努力扩大棚菜、优质水果、花生、花卉、食用菌等生产面积；提高新优品种比例，优质品种覆盖率达到９５％以上。大力发展绿色农业，积极开展绿色农产品生产基地的环评和无公害、有机、绿色农产品的认证。进一步加快水产品养殖业发展，水产品总量达到３２万吨以上。努力发展创汇农业，不断扩大农产品出口创汇份额。坚持用工业化理念谋划农业发展，积极发展农产品加工业，规模以上产业化企业达到１３５户，农产品加工销售收入达到４０亿元。

　　加快转变养殖方式。认真吸取禽流感教训，全面落实扶持家禽饲养业的各项措施，高标准做好农村养殖业发展规划，努力使养殖业向规模化、园区化、现代化方向发展。抓紧配齐配强监测、防疫人员，建立起完善的动物卫生防疫及监控体系，降低畜禽养殖风险，确保畜禽产品质量安全。新建标准化畜牧小区５０个，实现畜牧业产值９２亿元。

　　切实改善农村发展环境。认真贯彻落实党在农村的土地延包、＂两减免三补贴＂和农村税费改革的各项政策。加大财政对农业和农村的支持力度，集中城市优势资源，重点帮促经济薄弱乡村。统筹城乡义务教育和公共卫生等社会事业发展，逐步缩小城乡差距。加快推进大小凌河防洪和防海水入侵工程。依托＂三沿＂抓好＂四绿＂，完成人工造林面积３５万亩和大凌河口湿地保护区建设工作。全面实施＂兴机惠农＂工程，农业机械化水平达到５８％。加快农村剩余劳动力向非农产业和城镇转移，全年转移劳动力５万人，努力增加农民现金收入。认真做好全国第二次农业普查的前期准备工作。

　　（四）完成南扩十大工程，提高城市建设管理水平

　　推进城市南扩工程。渤海大道８月底前竣工通车，市府广场８月底前交付使用，女儿河带状公园　１０月底前完成改造，南山核心区绿化全面完成，市第三医院正式挂牌，凌南东里、西里等地段房地产开发全面启动，科技路、市府东路等路网全面贯通，滨海公路建设抓紧推进，确保三年的城市南扩任务两年基本完成。

　　加大城市管理力度。坚持建管并举，加快环卫、城管体制改革进程，逐步建立城市长效管理机制。严看死守、严管重罚，进一步巩固门前三包和网格化管理成果，坚决维护城市规划管理的严肃性和连续性。治理城乡环境，搞好城市建筑垃圾处理和危险废物综合利用工程建设，严厉查处环境违法案件。深入开展全国卫生城和环保模范城创建工作。积极稳妥地推进供暖体制改革。做好土地储备整理与经营开发工作，实行市场化运作，公开拍卖一批土地使用权，进一步盘活土地资源。

　　加快老城区改造和重点工程建设步伐。开工建设汉口街公铁立交桥，完成小扒沟、白老虎屯明沟改暗渠工程；实施主城区街路照明改造工程，彻底消灭＂摸黑路＂；新建、改造科技路、松坡路等２０条主次干道，完成３５条小街小巷改造任务；加大城市绿化投入，新增绿地面积８５万平方米；全面完成城区非物业住宅区环境整治任务。切实搞好３００公里村通油路路基改造和省定１００公里、连通３４个村的村村通油路工程，加快推进高天地方铁路增能改扩建、疏港路大中修等项目进程，力争完成锦凌水库项目国家报批工作。积极争取政策性银行贷款、国债资金和上级拨款，多形式吸引社会资本参与城市基础设施建设。

　　（五）增强三产辐射功能，建设区域商贸中心

　　加速现代服务业发展。加快构筑物流业基础设施平台和公用信息平台，重点支持物流园区发展，尽早完成恒大物流和渤海物流二期工程建设，增强华联北方、宏基等物流企业的配送功能。加快会展、通信网络、中介信息等现代服务业的发展。新型业态销售额实现２６亿元，增长１５％。促进住房、汽车、教育等消费健康发展。

　　扩大商贸流通业规模。加强同域外服务贸易企业的合作，加速批发市场改造升级，做强光彩大型家居建材中心、宏发建材商城等交易方式现代、服务功能完备的批发市场，增强对辽西、蒙东乃至更大范围的辐射作用。积极引进沃尔玛、家世界等国内外知名企业，加快商业区街改造和社区服务网点建设。推进＂万村千乡＂市场工程，建设改造乡级农家店２０个，村级农家店９０个。

　　促进金融保险业繁荣。推进金融信用体系建设，强化金融监管，改善企业融资环境，支持金融机构扩大信贷投放，积极引进外埠银行进入我市，加强与国家开发银行以及其它银行的合作。充分发挥资本市场融资功能，扩大直接融资规模。重点抓好汉拿电机、沈宏实业等７家企业的拟上市工作。依法运用保险资金，进一步加快保险业发展步伐。

　　提高旅游业发展水平。按照＂夯实基础、叫响品牌、规范服务、强化管理、做大产业＂的工作思路，继续推进旅游基础设施开发建设；加大旅游宣传促销力度，全方位开拓旅游客源市场；强化行业管理，严格规范旅游市场秩序；全面整合旅游资源，建设京辽通廊黄金旅游线，重点推出红色旅游、绿色旅游等特色文化旅游项目。旅游总收入增长２０％以上。

　　（六）用足用好开放政策，实现招商引资突破

　　抓住重要机遇，争取政策支持。学深学透国办３６号文件，用足用好２９条开放政策。进一步完善锦州湾整体开发规划，做好相应政策衔接工作，力争在港口建设、开发区建设、重大项目和重点基础设施建设等方面得到国家和省的政策支持。

　　注重项目包装，创新招商方式。确保每个县（市）区　＂精品＂　包装项目不少于５个，全市不少于１００个。加大在谈项目跟踪服务力度，加快阿迪达斯运动鞋、路易达孚大豆深加工等项目的落户进程。加强与香港贸发局、台湾中小企业经贸策进会等行业协会的合作，切实发挥行业协会的中介作用，拓宽招商引资市场化运作渠道。注重招商引资实效，重点组织好针对韩国、日本、珠三角、长三角、港澳台等国家和地区的小规模、多批次招商引资活动。

　　扩大开放领域，优化投资环境。全面放开产权、经营、作业市场，力争城市基础设施建设、公用事业、房地产、旅游等领域在利用外资上取得实质性突破。沉淀部分经济增量收入建立＂开放基金＂，支持园区发展和入园企业建设；逐步取消省以下收费项目，严格控制收费标准；积极利用开发区特殊管理权限，减少项目审批环节，缩短项目审批时间。进一步优化出口商品结构，努力扩大外贸出口份额。

　　（七）努力增加财税收入，壮大县域经济实力

　　切实增强财税增收能力。坚持把培植税源型企业作为财源建设的发展方向，逐步提高经济发展对财政收入的贡献率，经济指标对财政收入的转化率，实现财政收入与生产总值的协调增长，切实提高政府可支配财力规模。加强重点税源和税种的监控，加大税收稽查力度，挖掘增收潜力，大力清理欠税，坚决做到应收尽收。严格界定税费优惠条件，严厉打击和制止各种＂拉税＂以及违背国家政策的＂先征后返＂等行为。大力压缩一般性支出，确保公教人员工资按时足额发放和社会保障资金需要，重点保证农业、教育、科技等法定支出。加强对财政资金运行全过程的审计监督，严肃财经纪律。

　　提高县域经济发展能力。各县（市）区要积极围绕主导产业加快发展，进一步调整优化税源结构；尽快消除禽流感恢复期内不准养殖家禽等滞后影响，积极引导农户利用现有场所进行转产；大力扶持发展产业关联度高、经营机制活的中小企业，加速引进大企业、大项目，力争早投产、早达效、早获益；依托和发掘自身优势，着力培育工业、贸易企业向集群化发展；推进沟帮子开发区建设，更好发挥其对地区经济发展的拉动作用；加大双羊、大虎山、七里河、营盘等各类工业园区、工业小区的资源整合力度，提高园区土地利用效益。

　　（八）大力发展社会事业，积极打造文化名城

　　致力科技创新，加快教育发展。加大科技投入力度，积极发展电子信息、生物医药、新型材料等高新技术产业；提高企业自主创新能力，有效保护知识产权，进一步加快科研成果的转化和利用。全市高新技术产业产值增长２５％以上。实施人才强市战略，强化人才资源的市场化配置，大力培养和引进经济社会发展急需的高层次、高技能人才。全面推进素质教育，加强改进德育工作。抓好中小学教师岗位培训，深化课程改革，巩固提高＂普九＂成果。搞好危旧校舍改造，落实好农村义务教育＂两免一补＂的要求。推进机电工程学校搬迁和职业学校布局调整，加大中等职业教育招生规模，进一步提升职业教育水平。

　　全力办好省十运会，活跃群众文化生活。抓紧做好省十运会筹备工作，高标准完成场馆及相关设施建设；精心组织好开幕式、闭幕式和接待工作；提高运动成绩，确保实现金牌总数、奖牌总数、团体总分＂保五争四＂目标。举办好古塔庙会、北方农展会、笔架山海会、国际民间文化节等大型群众文化活动，积极吸引群众的广泛参与；修改加工《酒魂》等重点剧目，力争进入全省文化精品工程行列；抓好乡镇、社区等文化中心创建工作，夯实群众文化基础；继续深化文化体制改革，不断壮大文化产业。加大投入，出台政策，启动广播电视数字化整体平移工程，完成广播电视＂村村通＂工程。加强文物和非物质文化遗产的保护工作，进一步做好医巫闾山、北镇庙、奉国寺等＂申遗＂工作。

　　完善公共卫生体系，做好人口计生工作。推进１９所乡镇中心卫生院、６所偏远卫生院规划建设，启动试行涉农县（市）区的新型合作医疗制度；大力发展社区卫生服务，覆盖率达到９０％；加大药品流通改革力度，建立完善惠民医疗制度。全面落实农村６０岁以上独生子女户、双女户父母奖励扶助政策，全市人口出生率和自然增长率分别控制在８．４‰和３．４‰以内。

　　（九）切实关心百姓生活，全力构筑和谐锦州

　　做好就业再就业工作。进一步落实就业再就业扶持政策，完善就业再就业责任体系，大力开展就业援助，解决＂零就业家庭＂和大龄就业群体就业难问题。积极推行普惠制培训，着力提高下岗失业人员的职业技能和就业本领。实现实名制就业６万人，其中安置大龄就业困难人员４０００人；实施普惠制培训２万人，劳务输出３万人。加大劳动合同管理和劳动监察执法力度，切实维护劳动者合法权益。

　　完善社会保障体系。强化基金征缴和监管，依法扩大各项社会保险覆盖面。养老、失业、医疗、工伤、生育保险人数分别达到３２万人、４０万人、４１万人、２２万人、９万人。确保离退休人员养老保险金按时足额发放。开通社会保障网站和＂１２３３３＂劳动保障咨询服务电话，加快企业退休人员社会化管理步伐，推进社会保障信息化建设。

　　健全社会救助体系。强化城市低保规范化管理，稳步推进农村低保制度的实施。完善城乡医疗救助体系，解决看病难、看大病更难的问题。发挥慈善总会作用，采取政策扶贫、科技扶贫措施，妥善解决弱势群体尤其是禽流感疫区受灾群众的生产生活问题；做好残疾人康复、就业、维权等项工作；落实好贫困户三年建房规划。加快老年公寓等社会福利设施建设，改善农村敬老院条件。做好第六轮全国双拥模范城评比的准备工作。加强社区基础设施建设，提高社区的服务功能。

　　全力维护社会稳定。保持高压态势，严厉打击各种社会黑恶势力和凶杀绑架等严重刑事犯罪。建立健全社会安全预警机制，加强社会治安综合治理，增强城乡居民的安全感。认真做好群众来信来访、人民调解工作，努力化解社会矛盾。推进基层司法所建设，切实搞好普法工作。加强国家安全工作，全力维护社会政治稳定。落实安全生产责任制，加大安全生产执法力度，消除各种事故隐患。强化食品卫生监督，实施＂放心菜＂、＂放心肉＂工程；加大对生产、销售假冒伪劣商品等违法犯罪活动的打击力度，维护人民群众切身利益。

　　（十）增强行政执行能力，树立政府崭新形象

　　求真务实，恪尽职守。牢固树立正确的政绩观，脚踏实地、实事求是，不讲空话、不图虚名，严禁虚报浮夸、不搞形象工程。自觉接受人大及其常委会的法律监督、工作监督和人民政协的民主监督，认真执行人大关于政府工作的各项决议、决定，周密安排人大代表和政协委员的视察活动，切实提高人大议案、代表建议和政协提案的办理质量。加强同民主党派、工商联、无党派人士联系，集思广益，更好履行政府工作职责。

　　雷厉风行，高效行政。着力建设反应快速、高效运转的行政指挥系统，加快推进法治型、服务型、公信型、实干型政府建设进程。深入贯彻《全面推进依法行政实施纲要》、《行政许可法》，严格依法行政。推行行政执法责任制，认真做好行政复议工作，坚决纠正违法行政行为。规范政府职能定位，不断提高政府服务水平。大力推行政务公开，进一步增加政府工作透明度。强化政务督察工作，确保各项工作落到实处。

　　令行禁止，清正廉明。全力维护政令的权威性，切实增强政令的执行力。严格执行《关于严肃机关工作纪律的四项规定》等规章制度，对违规者严惩不贷。认真贯彻《建立健全教育、制度、监督并重的惩治和预防腐败体系实施纲要》，全面落实市委具体实施意见，严肃查处领导机关和领导干部滥用权力、谋取非法利益的案件，坚决惩处腐败分子。切实加强廉政自律教育，坚决反对和制止奢侈浪费。

　　严细成风，勤政为民。大力倡导严格严谨、仔细缜密的工作作风，进一步强化敢于负责、甘于奉献的为民理念。积极稳妥地推进行政事业单位改革，加快政府职能转变步伐。坚持和完善重大事项新闻发布制度，保证群众的知情权、参与权和监督权。不断拓宽政府与群众的沟通渠道，广泛听取各方面意见，真正实现好、维护好、发展好人民群众的根本利益。

　　各位代表，人民群众对政府的信任和支持，给了我们无穷的力量和必胜的信心。我们将在市委的坚强领导下，埋头苦干，奋力拼搏，确保今年全市各项工作的顺利推进，努力为＂十一五＂　高起点开局和＂三年大变样＂目标的实现做出更大的贡献！
